# Numerical Homogenization of Multi-Layered Corrugated Cardboard with Creasing or Perforation

**DOI:** 10.3390/ma14143786

**Published:** 2021-07-06

**Authors:** Tomasz Garbowski, Anna Knitter-Piątkowska, Damian Mrówczyński

**Affiliations:** 1 Department of Biosystems Engineering, Poznan University of Life Sciences, Wojska Polskiego 50, 60-627 Poznań, Poland; tomasz.garbowski@up.poznan.pl; 2 Institute of Structural Analysis, Poznan University of Technology, Piotrowo 5, 60-965 Poznań, Poland; 3 Research and Development Department, Femat Sp. z o. o., Romana Maya 1, 61-371 Poznań, Poland; damian.mrowczynski@fematproject.pl

**Keywords:** corrugated cardboard, numerical homogenization, strain energy equivalence, perforation, creasing, flexural stiffness, torsional stiffness

## Abstract

The corrugated board packaging industry is increasingly using advanced numerical tools to design and estimate the load capacity of its products. This is why numerical analyses are becoming a common standard in this branch of manufacturing. Such trends cause either the use of advanced computational models that take into account the full 3D geometry of the flat and wavy layers of corrugated board, or the use of homogenization techniques to simplify the numerical model. The article presents theoretical considerations that extend the numerical homogenization technique already presented in our previous work. The proposed here homogenization procedure also takes into account the creasing and/or perforation of corrugated board (i.e., processes that undoubtedly weaken the stiffness and strength of the corrugated board locally). However, it is not always easy to estimate how exactly these processes affect the bending or torsional stiffness. What is known for sure is that the degradation of stiffness depends, among other things, on the type of cut, its shape, the depth of creasing as well as their position or direction in relation to the corrugation direction. The method proposed here can be successfully applied to model smeared degradation in a finite element or to define degraded interface stiffnesses on a crease line or a perforation line.

## 1. Introduction

Colorful boxes and packaging are designed to attract the customers’ attention and, as a consequence, to drive the sales of various goods ranging from bulky products, through food, children’s toys, cosmetics, and many others. A growing awareness of concern for the natural environment has led many companies to opt for packaging that can be easily recycled or disposed of, biodegradable, and space-saving after manufacturing. A corrugated cardboard undoubtedly has all of these qualities. Moreover, it is easy to print on, for example, the brand name. Corrugated cardboard is easy to shape via creasing along the suitable lines and, furthermore, creating openings, ventilation holes, or perforations does not cause much difficulty. The latter is essential with regard to shelf-ready packaging (SRP) or retail-ready packaging (RRP) when the product, after transportation to the site, is placed on the shelves and after tearing off the flap along the appropriately designed perforation, is ready for sale. Thus, a lot of time is saved, which nowadays leads to significant profits for large companies.

Of course, one cannot only focus on the aesthetic values because the packaging, in fact, plays a much more important role such as securing the goods during storage or safe transport to the destination place. The load-bearing capacity of the corrugated cardboard boxes and the influence of humidity, openings and perforation arrangement, or the location of flaps is under constant investigation. Therefore, scientific research has become an integral part of a distinct branch of industry (i.e., cardboard packages production). Manufacturers of these packaging types strive for effective, economical, and easy-to-use solutions, which results in the continuous, lasting over many years, development of research on cardboard strength while using various analytical, numerical, and experimental methods.

Compressive, tensile, or bursting strength tests are routinely executed to assess the load-bearing capacity of corrugated cardboard boxes. The box compression test (BCT) and the edge crush test (ECT) are the best known. Inextricably related to the mechanical strength of the paperboard or corrugated cardboard boxes are two characteristic in-plane directions of orthotropy (i.e., perpendicular to the main axis of the fluting and parallel to the paperboard fiber alignment—machine direction (MD) as well as parallel to the fluting—cross direction (CD)).

Another option for estimating the compressive strength of the boxes is the application of analytical formulae in which, in general, three groups of parameters such as paper, board, and box parameters are present [1]. Ring crush test (RCT), Concora liner test (CLT), liner type, weights of liner and fluting, corrugation ratio, and a constant related to fluting belong to the first group. Thickness, flexural stiffnesses in MD and CD, ECT, and moisture content are affiliated with the second group whereas dimensions and perimeter of the box, applied load ratio, stacking time, buckling ratio, and printed ratio are in the third one. Already in 1952, Kellicutt and Landt [2] proposed the calculations of box compressive strength while employing the formula with parameters introduced in the paper (RCT, flute constant) and box (perimeter, box constant). In 1956, Maltenfort [3] indicated the relation between the critical force and paper parameters (CLT, type of liner) and cardboard box dimensions in the BCT. In the approach proposed by McKee, Gander, and Wachuta [4] in 1963, the parameters of the paperboard (ECT, flexural stiffnesses) and the box perimeter were applied. Even though this formula is commonly used in the packaging industry due to its simplicity, which leads to quick and easy solutions for practical implementations, it is applicable only to simple standard boxes. Therefore, scientists have been making attempts to extend the implementation of McKee’s analytical approach. Allerby et al. [5] modified the constants and exponents, whilst Schrampfer et al. [6] improved McKee’s method by expanding the range of cutting methods and equipment. Batelka et al. [7] augmented the relationship by introducing the dimensions of the box and Urbanik et al. [8] included the Poisson’s ratio. Further modification of the above-mentioned McKee’s formula for solving more complex problems has been proposed by Aviles et al. [9] and later, by Garbowski et al. [10,11,12].

Over recent decades, meshless and meshfree methods (e.g., the collocation method) have become popular numerical techniques for solving partial differential equations and have been beneficial while considering corrugated cardboard problems. Wang and Qian [13] proposed the meshfree stabilized collocation method (SCM) and introduced the reproducing kernel function as the approximation. Wang et al. [14] employed the meshfree radial basis collocation method (RBCM), which utilizes infinitely continuous radial basis functions (RBFs), as the approximation for the static and dynamic eigenvalue analysis of the thin functionally graded shells (FGSs) with in-plane material inhomogeneity. The buckling analysis of thin FG plates, also with in-plane material inhomogeneity, while applying radial basis collocation method (RBCM) and Hermite radial basis function collocation method (HRBCM) was discussed by Chu et al. [15]. The main advantages of the above-mentioned approaches are high accuracy and exponential convergence.

Unquestionably, many determinants affect the compression strength of the corrugated paperboard boxes [16] including the moisture content of the box [17,18], openings, ventilation holes and perforations [11,12,19], storage time and conditions [20], stacking load [21], or a very significant one—creasing. As a result of such a process, fold and perforation lines are performed and through this, the mechanical strength of the manufactured corrugated paperboard boxes is diminished.

A very effective, commonly applied in engineering, technique to determine the strength of the boxes is the finite element method (FEM). Thakkar et al. [22] compared the experimental and FEM numerical results to investigate the creasing impact on the local strength of corrugated paperboard; Beex and Peerlings [23], in turn, conducted physical and numerical experiments to examine the influence of creasing and subsequent folding on the mechanical properties of the laminated paperboard. A constitutive model was implemented by Giampieri et al. [24] in order to obtain the mechanical response of creased paperboard after folding. FEM simulations of paperboard creasing, which appeared to be significant from a practical standpoint, have been proposed by Domaneschi et al. [25] and Awais et al. [26]. Leminena et al. [27] performed experimental and numerical analyses to examine the influence of the creasing process during the press forming on the paperboard mechanical properties. FEM has also been involved in research raising the issue of numerical analysis in relation to transverse shear stiffness of the corrugated cardboards [28,29,30,31,32] or buckling and post-buckling phenomena [33].

The examined models can be facilitated to one single layer described by the effective properties of the composite instead of building layers composed of different materials. Such a method, called homogenization, has been used extensively over the last years by Garbowski et al. [32,34,35,36,37]. A clear advantage of this technique is the significant saving in calculation time while preserving the precision of the results. Hohe [38] proposed a representative element of the heterogeneous and homogenized elements based on strain energy to analyze sandwich panels. A periodic homogenization method presented by Buannic et al. [39] enabled them to obtain an equivalent membrane and pure bending characteristics of period plates and, in a modified version, to incorporate the transfer shear effect in the analysis. Biancolini [40] engaged FEM to study a micromechanical part of the considered plate. Thanks to the energy equivalence between the model and the homogenized plate, the stiffness properties of the sandwich plate were received. Decomposition of the plate into two beams in directions of the plate allowed Abbès and Guo [41] to define the torsion rigidity of the orthotropic sandwich plates. An interesting approach based on empirical observation can also be found in the recent work of Gallo et al. [21]. A multiple scales asymptotic homogenization approach was presented by Ramírez-Torres et al. [42] where the effective properties of hierarchical composites with periodic structure at different length scales has been studied, whereas in [43], the authors used the asymptotic homogenization technique to the equations describing the dynamics of a heterogeneous material with evolving micro-structure, obtaining a set of upscaled, effective equations.

The following article, as the next one in the series, provides theoretical considerations that develop and extend the numerical homogenization technique already presented in the prior works of the authors. The proposed homogenization procedure also takes into consideration the creasing and/or perforation of corrugated board (i.e., processes that evidently weaken the stiffness and strength of the corrugated board locally). However, it is not always easy to estimate how exactly these processes affect the bending or torsional stiffness. The fact is that the decrease in stiffness depends, among others, on the type of cut, its shape, and the depth of creasing as well as their position or direction in relation to the corrugation orientation. The method proposed here can be successfully implemented to model smeared degradation in a finite element or to define degraded interface stiffnesses on a crease line or a notch line.

## 2. Materials and Methods

### 2.1. Corrugated Board—Material Definition

**Figure 1:**
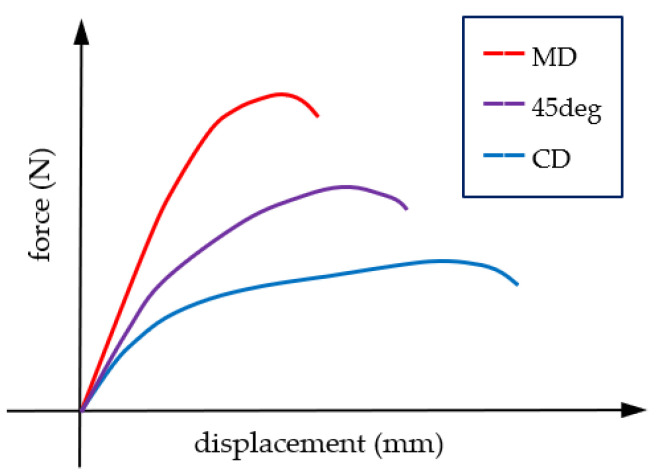
Paperboard mechanical behavior. The stress–strain relationships in different material directions.

Corrugated board, as a fibrous material, is characterized by strong orthotropy. The mechanical properties of its components (i.e., cardboard) depend on the direction of the fibers in the individual layers of the composite. Paper and paperboard are more than twice as stiff in the machine direction (MD) than in the cross direction (CD). This is related to the fibers which, due to the production process, arrange along the MD. In this direction, the material is more resistant to tearing and crushing, although it has lower ductility than in CD (see Figure 1).

The linear elastic orthotropic material can be described by the following stress–strain relationships:

(1) $$\left[\begin{array}{c} \varepsilon_{11}\\ \varepsilon_{22}\\ 2\varepsilon_{12}\\ 2\varepsilon_{13}\\ 2\varepsilon_{23} \end{array}\right]=\left[\begin{array}{ccccc} 1/E_{1} & -\nu_{21}/E_{2} & 0 & 0 & 0\\ -\nu_{12}/E_{1} & 1/E_{2} & 0 & 0 & 0\\ 0 & 0 & 1/G_{12} & 0 & 0\\ 0 & 0 & 0 & 1/G_{13} & 0\\ 0 & 0 & 0 & 0 & 1/G_{23} \end{array}\right]\left[\begin{array}{c} \sigma_{11}\\ \sigma_{22}\\ \sigma_{12}\\ \sigma_{13}\\ \sigma_{23} \end{array}\right]$$

where $E_{1}$ is the Young’s modulus in the machine direction (MD); $E_{2}$ is the Young’s modulus in the cross direction (CD); $G_{12}$ is the Kirchhoff’s modulus, $\nu_{12}$; $\nu_{21}$ is the Poisson’s coefficients. Due to the symmetry of the material compliance/stiffness matrix, the relationship between the Poisson’s coefficients is as follows:

(2) $$\frac{\nu_{12}}{E_{1}}=\frac{\nu_{21}}{E_{2}}$$

**Figure 2:**
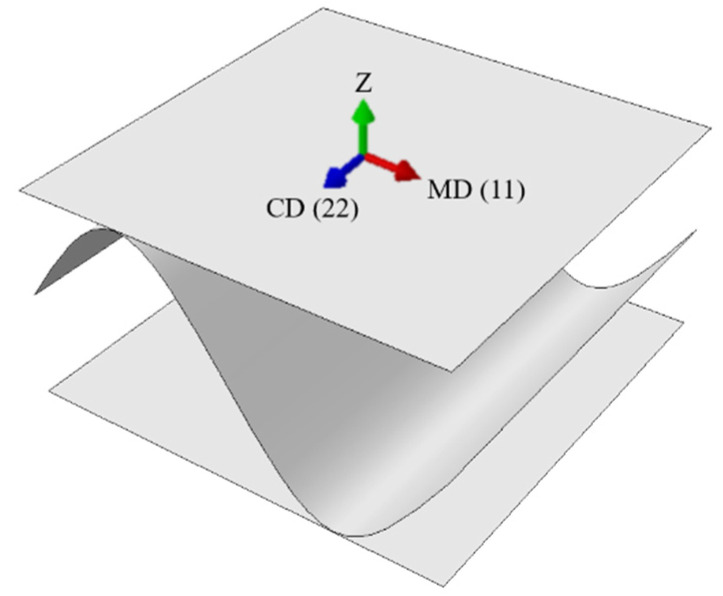
Material orientation.

The material orientation was always the same in all layers (see Figure 2). This is related to the corrugated board production process in which the paper (for the production of both flat and corrugated layers) is rolled on a corrugator machine from multi-tone bales.

The paperboard, as already mentioned, was modeled here using classical linear elastic orthotropy (see Equation (1)). The material data were taken from the literature [40,44,45]. All material data are presented in Table 1 (i.e., $E_{1}$, $E_{2}$, $v_{12}$, $G_{12}$, $G_{13}$ and $G_{23}$, which represents Young’s moduli in both directions, Poisson’s ratio, in-plane shear modulus and two transverse shear moduli, respectively).

The thickness of all flat layers (liners) in both single- and double-walled corrugated boards was assumed to be 0.30 mm; for all corrugated layers (flutes) in both models, the thickness was also taken as 0.30 mm.

### 2.2. Creases and Perforations—Numerical Study

**Figure 3:**
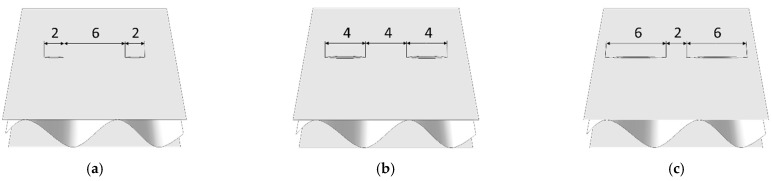

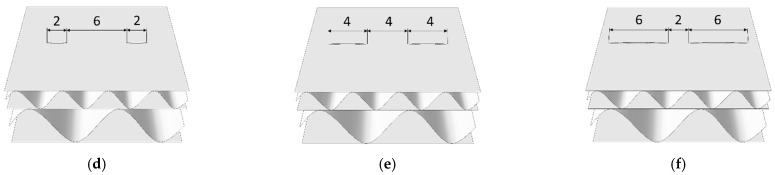
Perforation types: (**a**) Type 2/6—model SW; (**b**) Type 4/4—model SW; (**c**) Type 6/2—model SW; (**d**) Type 2/6—model DW; (**e**) Type 4/4—model DW; (**f**) Type 6/2—model DW.

The main goal of this work was to numerically analyze many cases of perforation with possible creasing and its effect on the stiffness reduction of corrugated board. The variants include not only different types of perforation (e.g., 4/4—4 mm cut, 4 mm gap; 2/6—2 mm cut, 6 mm gap; and 6/2—6 mm cut, 2 mm gap), but also different orientations of the cuts in the sample (from 0 to 90 deg. every 15 degrees). All cases are compiled in Table 2 and are shown in Figure 3.

**Figure 4:**
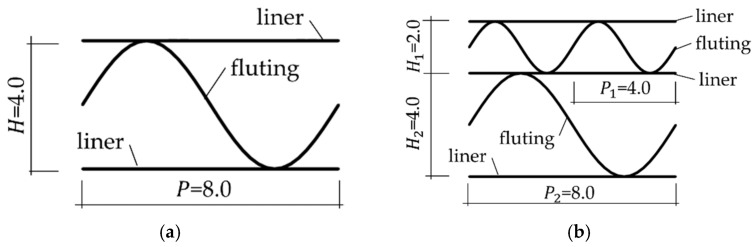
Geometry of the sample: (**a**) single layer; (**b**) double layer.

Two hypothetical corrugated boards were analyzed here, namely single-walled (SW) with 8 mm flute period, 4 mm height and double-walled (DW) with 4 mm flute period, 2 mm flute height (for lower layer) and 8 mm flute period, 4 mm flute height (for higher layer). Figure 4 shows the visualizations of the geometry of both examples.

**Figure 5:**
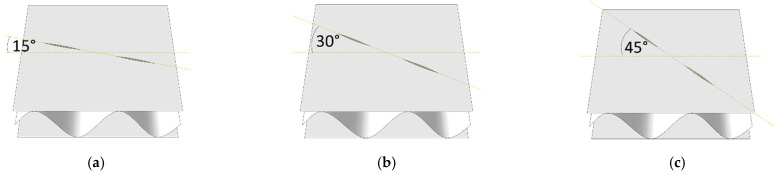

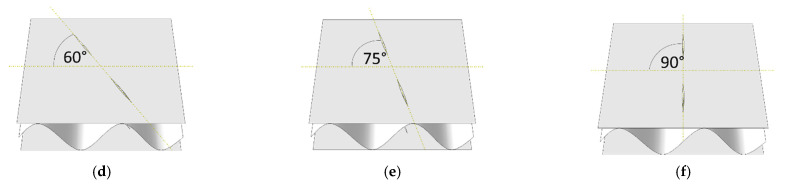
Perforation orientation in sample SW-44-C: (**a**) rotation by 15 degrees; (**b**) rotation by 30 degrees; (**c**) rotation by 45 degrees; (**d**) rotation by 60 degrees; (**e**) rotation by 75 degrees; (**f**) rotation by 90 degrees.

Both the influence of the flute orientation and the cutting orientation on the decrease in the stiffness of the corrugated board were examined. In case C, the cutting orientation changed to 00, 15, 30, 45, 60, 75, 90 degrees (see Figure 5) while the flute orientation remained constant.

**Figure 6:**
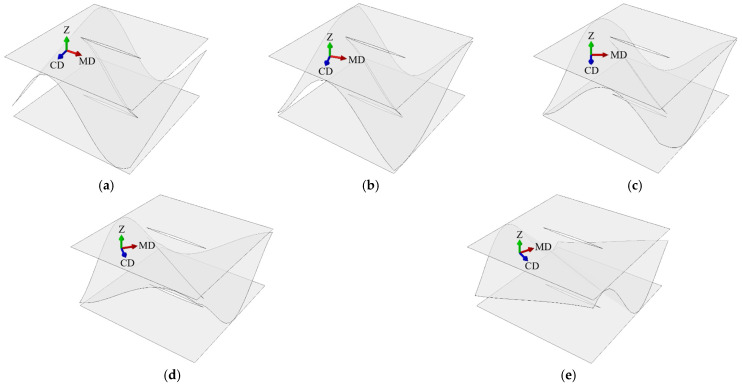
Perforation orientation in sample SW-44-F: (**a**) rotation by 15 degrees; (**b**) rotation by 30 degrees; (**c**) rotation by 45 degrees; (**d**) rotation by 60 degrees; (**e**) rotation by 75 degrees.

**Figure 7:**
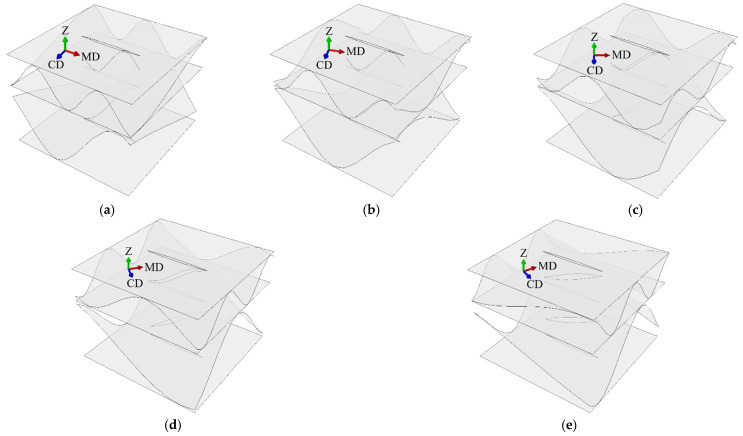
Perforation orientation in sample DW-44-F: (**a**) rotation by 15 degrees; (**b**) rotation by 30 degrees; (**c**) rotation by 45 degrees; (**d**) rotation by 60 degrees; (**e**) rotation by 75 degrees.

In case F, the flute orientation were changed to 00, 15, 30, 45, 60, 75, 90 degrees (see Figure 6 and Figure 7) while the cut orientation remained constant. All cases are summarized in Table 2.

**Figure 8:**
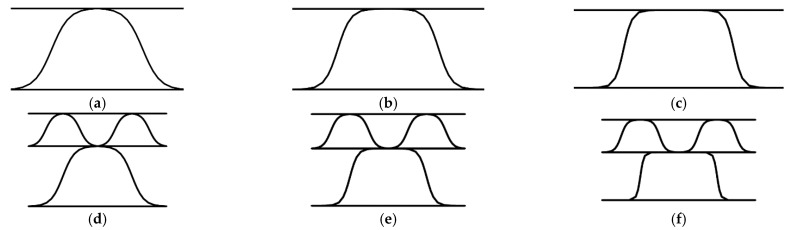
Crushed samples: (**a**–**c**) Single-walled sample crushed by 10%, 20%, and 30%, respectively; (**d**–**f**) Double-walled sample crushed by 10%, 20%, and 30%, respectively.

Both single-walled and double-walled models with perforations of 4/4 mm, 2/6 mm, and 6/2 mm in the variant 00 deg. of cut and flute rotation were crushed by 10, 20, and 30%. This consideration results from the observation of the serial production of packaging in which crushing is an element built into the entire cutting and perforation process. The additional crushing during cutting is the result of using rubber in the area of perforation knives that additionally crush the cross-section. The crushed geometry of both kinds of samples is shown in Figure 8.

All crushed samples were marked with an additional symbol R-xx, where xx means the amount of crush (i.e., 10, 20, or 30). Therefore, for example, a single-walled specimen with a cut/flute rotated by 0 degrees with a cut version of 44 and crushed by 10% has the symbol SW-44-C-00-R-10.

**Figure 9:**
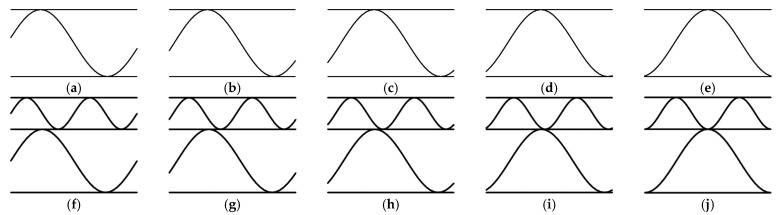
Cross section of the corrugated board along the wave: (**a**) the reference SW sample—no offset; (**b**) SW sample—offset equal to 1/16 P; (**c**) SW sample—offset equal to 2/16 P; (**d**) SW sample—offset equal to 3/16 P; (**e**) SW sample—offset equal to 4/16 P; (**f**) the reference DW sample—no offset; (**g**) DW sample—offset equal to 1/16 P; (**h**) DW sample—offset equal to 2/16 P; (**i**) DW sample—offset equal to 3/16 P; (**j**) DW sample—offset equal to 4/16 P.

Additionally, what was verified during this research was the influence of the position of the cut in the corrugated boards’ cross-section along the wave on the stiffness reduction. For this purpose, four additional representative volumetric element (RVE) models were created in two variants of the SW and DW samples, in which the flute was shifted by 1/16 of the period (P) from 1/16 P to 4/16 P (see Figure 9).

### 2.3. Homogenization Technique

In order to determine the effect of cuts on the stiffness of the corrugated board, the numerical homogenization method was used here. This method, originally proposed by Biancolini [40] and later extended by Garbowski and Gajewski [32], is based on the elastic energy equivalence between the simplified shell model and the full RVE of corrugated cardboard. The RVE is a finite element (FE) representation of a small, periodic section of the full 3D corrugated board structure. The complete derivations of the constitutive model can be found in [32]. In the present study, only the basic assumptions are presented below.

**Figure 10:**
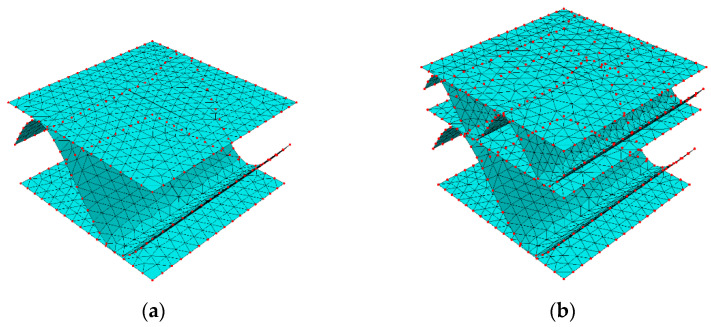
RVE—external (in red color) and internal nodes and finite elements: (**a**) SW model; (**b**) DW model.

The displacement based on finite element formulation for a linear analysis can be represented by an equation:

(3) $$K_{e}\;u_{e}=F_{e},$$

where $K_{e}$ is a statically condensed global stiffness matrix of the RVE; $u_{e}$ is a displacement vector of external nodes; and $F_{e}$ is a vector of the nodal forces applied to external nodes. In Figure 10, the FE mesh and mesh nodes are shown.

Static condensation relies on the removal of unknown degrees of freedom (DOF) and then the formulation of the stiffness matrix for a smaller number of degrees of freedom, called the primary unknown or principal DOF. In the analyzed cases, the eliminated degrees of freedom is the internal RVE nodes and the external nodes are the primary unknowns. The statically condensed FE stiffness matrix is computed from the equation:

(4) $$K_{e}=K_{ee}-K_{ei}\;K_{ii}^{-1}K_{ie},$$

where the stiffness matrix contains four subarrays related to internal (subscript i) and external (subscript e) nodes:

(5) $$\left[\begin{array}{cc} K_{ee} & K_{ei}\\ K_{ie} & K_{ii} \end{array}\right]\left[\begin{array}{c} u_{e}\\ u_{i}\; \end{array}\right]=\left[\begin{array}{c} F_{e}\\ 0 \end{array}\right].$$

Static condensation reduces the total elastic strain energy to the work of external forces on the corresponding displacements. The total elastic strain energy can be calculated from the equation:

(6) $$E=\frac{1}{2}u_{e}^{T}\;F_{e}.$$

The balance of the total energy for the full 3D shell model and the simplified shell model is ensured by an appropriate definition of displacements in the external RVE nodes and by enabling the membrane and bending behavior. More details can be found in Garbowski and Gajewski [32]. The generalized displacements are related to the generalized strains on the RVE edge surfaces, which can be represented by the relationship:

(7) $$u_{i}=H_{i}\;\epsilon_{i},$$

where for a single node ($x_{i}=x$, $y_{i}=y$, $z_{i}=z$) the $H_{i}$ matrix adopted for RVE shell model can be determined:

(8) $${\left[\begin{array}{c} u_{x}\\ u_{y}\\ u_{z}\\ \theta_{x}\\ \theta_{y} \end{array}\right]}_{i}={\left[\begin{array}{cccccccc} x & 0 & y/2 & xz & 0 & yz/2 & z/2 & 0\\ 0 & y & x/2 & 0 & yz & xz/2 & 0 & z/2\\ 0 & 0 & 0 & -x^{2}/2 & -y^{2}/2 & -xy/2 & x/2 & y/2\\ 0 & 0 & 0 & 0 & -y & -x/2 & 0 & 0\\ 0 & 0 & 0 & x & 0 & y/2 & 0 & 0 \end{array}\right]}_{i}{\left[\begin{array}{c} \varepsilon_{x}\\ \varepsilon_{y}\\ \gamma_{xy}\\ \kappa_{x}\\ \kappa_{y}\\ \kappa_{xy}\\ \gamma_{xz}\\ \gamma_{yz} \end{array}\right]}_{i}$$

While using the definition of the elastic strain energy for a discrete model:

(9) $$E=\frac{1}{2}u_{e}^{T}\;K\;u_{e}=\frac{1}{2}\epsilon_{e}^{T}\;H_{e}^{T}\;K\;H_{e}\;\epsilon_{e}$$

and considering a finite element as subjected to bending, tension, and transverse shear, the elastic internal energy is expressed by:

(10) $$E=\frac{1}{2}\epsilon_{e}^{T}\;H_{k}\;\epsilon_{e}\left\{area\right\}.$$

For a homogenized composite, the stiffness matrix can be easily determined as:

(11) $$H_{k}=\frac{H_{e}^{T}\;K\;H_{e}}{area}.$$

The presented homogenization method is based on replacing the full 3D shell model with a simplified shell model and computing the effective stiffness of the RVE. Such a procedure significantly accelerates the computations and maintains a very high accuracy of the results.

The matrix $H_{k}$ is formed by the matrices **A**, **B**, **D**, and **R** as follows:

(12) $$H_{k}=\left[\begin{array}{ccc} A_{3\times 3} & B_{3\times 3} & \\ B_{3\times 3} & D_{3\times 3} & \\ & & R_{2\times 2} \end{array}\right]$$

where **A** represents extensional and shear stiffnesses; **B** represents extension-bending coupling stiffnesses; and **D** represents bending and torsional stiffnesses, while **R** represents transverse shear stiffness.

In general, the stiffness matrix A is independent of the position of a neutral axis. For the most symmetrical cross sections, all elements of stiffness matrix B are equal to zero. However, for unsymmetrical sections (i.e., double-walled corrugated board samples) matrix B is a non-zero, which indicates that there is a coupling between bending/twisting curvatures and extension/shear loads. Traditionally, these couplings have been suppressed for most applications by choosing the position of the neutral axis that minimizes the values of B. Alternatively, uncoupled matrix D can be computed from the formula:

(13) $$D=D_{0}-BA^{-1}B,$$

where $D_{0}$ represents the original (coupled) bending and torsional stiffnesses.

Within all analyses, the 3-node triangular general-purpose shell elements, named S3, were used for the computations. In every examined case, approximate global size equal to 0.5 mm was assumed. Due to the analysis of different orientations of flutings or cuts in the sample, the number of elements changed. For example, in the case of the SW-44-C-00 sample—2002 elements, 1099 nodes, and 6594 degrees of freedom were obtained, and for the DW-44-C-00 sample—3972 elements, 2074 nodes, and 12,444 degrees of freedom were obtained.

## 3. Results

### 3.1. Validation of the Proposed Method

**Figure 11:**
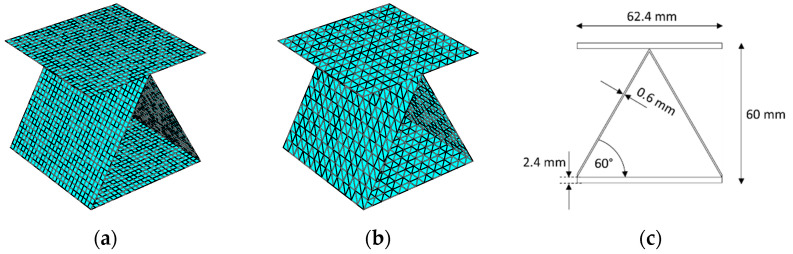
Representative shell elements of saw tooth geometry with quadrilateral mesh (single period): (**a**) model with a fine 4-node mesh; (**b**) model with a coarse 3-node mesh; (**c**) model geometry.

The proposed numerical method was first verified by direct comparison of the obtained results with the existing solutions from the literature. One example concerns an assembled sandwich structure consisting of a corrugated tooth-shaped core enclosed between two sheets. A reference solution is available from Buannic et al. [39]. According to the notation used in the literature, the T2 panel was tested here. The FE models used in this comparison for the T2 sandwich consists of 3-node and 4-node shell elements and are shown in Figure 11. Error estimation was performed and the maximum deviation was less than 2.5%.

On the basis of the above validation (see Table 3) carried out on two numerical models: (a) model with a fine mesh (see Figure 11a) and (b) model with a coarse mesh (see Figure 11b), it was found that the solution does not depend on the element type and on the size of the finite element. It is important, however, to correctly represent any curvatures, therefore, in the case of sinus-like fluting, at least 16 segments are required to obtain correct results [32].

### 3.2. Detailed Results

This section presents all the results of numerical tests for both single-walled (SW) and double-walled (DW) corrugated board samples. First, Table 4 and Table 5 show an example of the $A_{k}$ matrix, calculated while using the SW and DW models, respectively (both unperforated).

Due to the volume limitations of the data that can be presented in all the following tables, only the values from the main diagonals of the $A_{k}$ matrix are shown. This simplification does not introduce an error in the analyses of the results, mainly because the components ${\left(*\right)}_{12}$ are related to the elements ${\left(*\right)}_{11}$ and ${\left(*\right)}_{22}$ in each matrix. The B matrix was also disregarded. However, it has been accounted for using Equation (13) in the D matrix, which is presented in all tables below.

Since the DW model is asymmetric, all matrices **A**, **B**, **D**, and **R** are non-zero; in particular, matrix **B** (see Table 5), which combines the bending effects with the membrane stiffness of the plate.

Table 6 shows the selected stiffnesses of all SW models with no perforation and fluting, rotated by an angle of 0 to 90 every 15 degrees. It is worth noting that in the case of models with rotated fluting by 90 degrees SW-0-F-90 and with non-rotating fluting SW-0-F-0, the stiffness values ${\left(*\right)}_{11}$ and ${\left(*\right)}_{22}$ were swapped (the same holds for ${\left(*\right)}_{44}$ and ${\left(*\right)}_{55}$).

Table 7 shows the selected stiffnesses of all DW models with no perforation and fluting rotated by an angle of 0 to 90 every 15 degrees (see Figure 7). For the DW-0-F-45 and SW-0F-45 samples, the same values were obtained for all ${\left(*\right)}_{11}$ and ${\left(*\right)}_{22}$ as well as ${\left(*\right)}_{44}$ and ${\left(*\right)}_{55}$, which was expected. This is, of course, due to the symmetry in both the geometrical setup and the material orientation.

**Figure 12:**
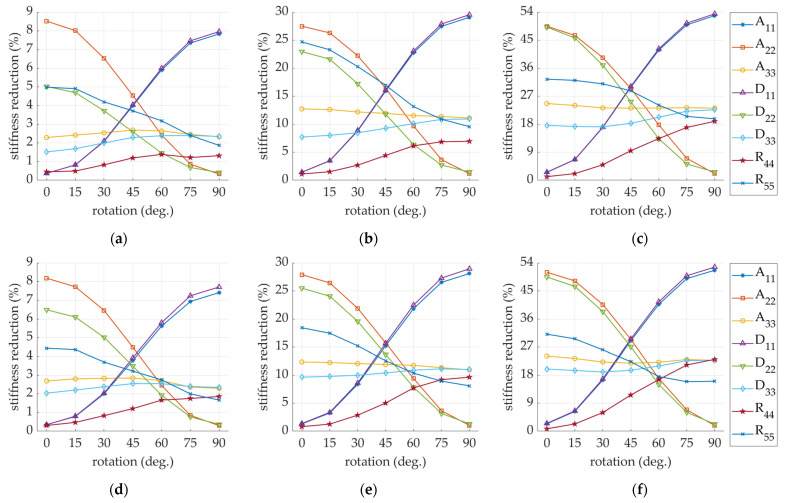
Stiffness degradation in sample: (**a**) SW-26; (**b**) SW-44; (**c**) SW-62; (**d**) DW-26; (**e**) DW-44; (**f**) DW-62.

Figure 12 shows the stiffness reduction of thee perforated models (both SW and DW) depending on the perforation rotation angle. The normalization term in each case is the $A_{k}$ matrix of the corresponding non-perforated sample (i.e., all stiffnesses in the perforated SW models are divided by the corresponding stiffnesses in nonperforated SW model).

Table 8 and Table 9 summarize the chosen values of stiffness for a selected case of SW sample with fluting rotated by 15 degrees, for four cases of perforation: (i) no perforation; (ii) 2/6 mm (i.e., the normalized cut is 25%); (iii) 4/4 mm (i.e., the normalized cut is 50%); and (iv) 6/2 mm (i.e., the normalized cut is 75%).

**Figure 13:**
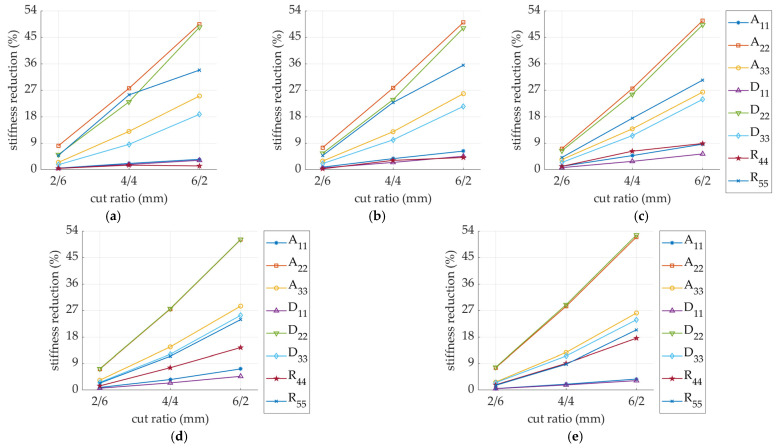
Stiffness degradation in sample SW: (**a**) F-15; (**b**) F-30; (**c**) F-45; (**d**) F-60; (**e**) F-75. Three types of perforations were analyzed (2/6 mm, 4/4 mm, or 6/2 mm).

**Figure 14:**
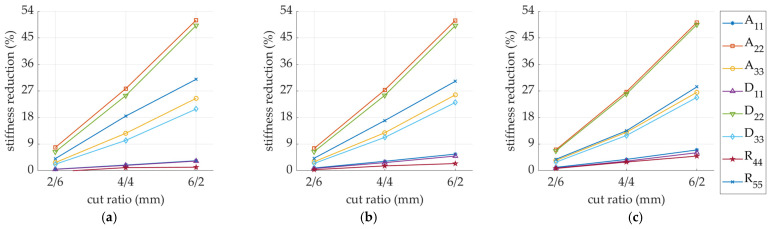

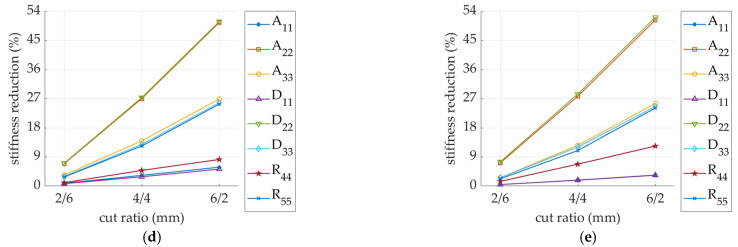
Stiffness degradation in a sample DW: (**a**) F-15; (**b**) F-30; (**c**) F-45; (**d**) F-60; (**e**) F-75. Three types of perforation were analyzed (2/6 mm, 4/4 mm, or 6/2 mm).

Figure 13 shows the selected values of the stiffness reduction of the SW samples with the flute rotated by 15, 30, 45, 60, and 75 degrees. All stiffnesses were normalized by the $A_{k}$ matrix of the non-perforated sample with the appropriate fluting orientation (see Figure 6). Figure 14 presents the selected values of the stiffness reduction of the DW samples with the flute rotated by 15, 30, 45, 60, and 75 degrees. All stiffnesses were normalized by the $A_{k}$ matrix of the non-perforated sample with the appropriate fluting orientation (see Figure 7).

**Figure 15:**
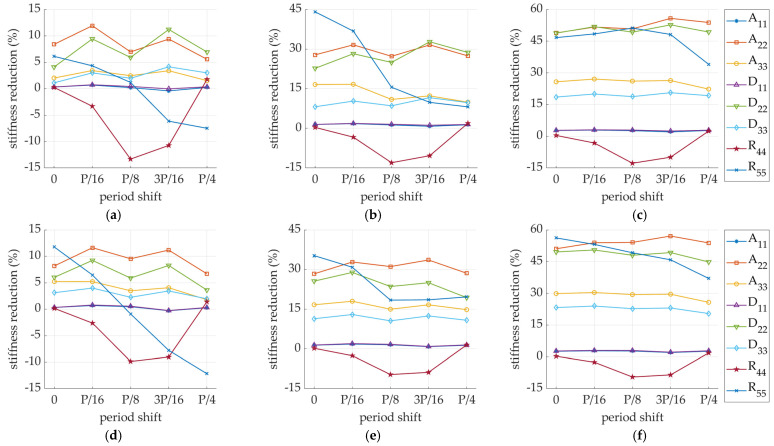
Stiffness degradation in sample C-0: (**a**) SW-26; (**b**) SW-44; (**c**) SW-62; (**d**) DW-26; (**e**) DW-44; (**f**) DW-62.

In the process of cutting corrugated board, perforation may occur in various locations relative to the fluting position, therefore the impact of fluting shift on stiffness changes has also been analyzed. Figure 15 presents the values of the stiffness reduction depending on the location of the cut in relation to the fluting position for the SW and DW samples in three perforation varieties: 2/6 mm, 4/4 mm, and 6/2 mm.

Due to noticed increase of $R_{44}$ and $R_{55}$ stiffnesses (negative stiffness reduction values shown in Figure 15), non-perforated samples were also examined. The values of the stiffness reduction depending on the fluting shift for the SW sample are summarized in Table 10, whereas the values of the stiffness reduction depending on the fluting shift for the DW sample are listed in Table 11.

**Figure 16:**
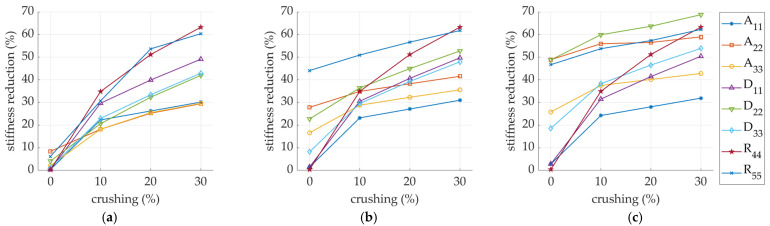

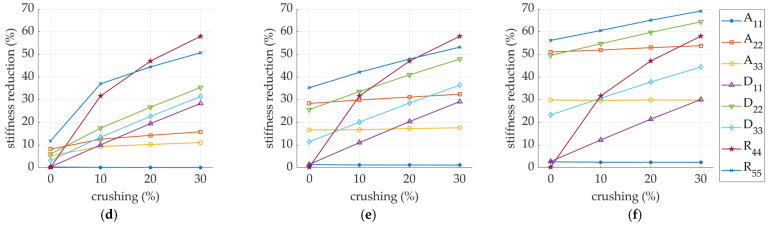
Stiffness degradation in sample: (**a**) SW-26-C-0-R-xx; (**b**) SW-44-C-0-R-xx; (**c**) SW-62-C-0-R-xx; (**d**) DW-26-C-0-R-xx; (**e**) DW-44-C-0-R-xx; (**f**) DW-62-C-0-R-xx. Here xx is a crush level (0%; 10%, 20%, and 30%).

As the perforation process is inseparable from the crushing process, this effect on the reduction of stiffness has also been tested. The influence of additional crushing of 10, 20, and 30% of the initial height of the corrugated board on the stiffness degradation of SW and DW samples is presented in Figure 16. The comprehensive study of the impact of crushing on single-walled corrugated board is presented in a recent study of Garbowski et al. [44], while for the double-walled structures, see Gajewski et al. [45].

## 4. Discussion

On the basis of the conducted analyses and the obtained results, it can be concluded that the perforations to a greater or lesser extent affected the stiffness degradation not only in the A sub-matrix (responsible for the tensile/compression stiffness) and in the D sub-matrix (responsible for bending/torsion stiffness), but also in the R sub-matrix (responsible for the transversal shear stiffness).

For samples with different perforation orientations (see Figure 5), the reduction in stiffness was related to the rotation angle of the perforation. In the samples with a rotation angle below 30 degrees, the greatest reduction occurred for matrix elements with indices 22 and 55. If the rotation angle was greater than 60 degrees, mainly matrix elements with indices 11 and 44 were reduced. This rule applied to both types of samples (i.e., SW and DW). When the perforation was rotated by an angle equal to 45 degrees, the matrix elements with indices 11, 22, 44, and 55 were evenly degraded.

For 2/6 mm perforation in model SW (see Figure 12a), the maximum degradation did not exceed 10% and was applied to $A_{22}$ (for perforation rotation angle < 30 degrees) and $A_{11}$, $D_{11}$ (for perforation rotation angle > 60 degrees). It is worth noting that the decrease in the stiffness $D_{22}$ and $R_{55}$ for the rotation angle of the perforation equal to 0 degrees was relatively high and amounted to 5% for the perforation type 2/6 mm. The remaining stiffnesses degraded less than 3% in this case. A similar observation applied to the DW model (see Figure 12d).

While considering the 4/4 mm type perforation (see Figure 12b), the observations were as follows: reduction of $A_{22}$, $D_{22}$ was about 25% for a perforation rotation of 0 degrees and about 0% for a 90-degree rotation; $R_{55}$ degraded about 25% when the perforation was rotated by 0 degrees and about 10% when the perforation was rotated by 90 degrees; reduction of $A_{33}$ and $D_{33}$ was about 10% regardless of the perforation rotation angle, while the degradation of $A_{11}$ and $D_{11}$ varied from around 0% to 30% for 0 degrees and 90 degrees, respectively; and the degradation of $R_{44}$ did not exceed 5%. In the DW model (see Figure 12e), a similar decrease could be observed. The reductions $R_{44}$ and $R_{55}$ look slightly different; this is related to a different ratio of the sample height to its dimensions in the plan.

The greatest reductions were observed for the sample with the 6/2 mm perforation type (see Figure 12c,f). This is obviously related to the largest cut-to-gap ratio (which amounts to 75% in this case). In the case of the SW model, both the stiffness reductions $A_{11}$ and $D_{11}$ as well as $A_{22}$ and $D_{22}$ reached a maximum value of slightly more than 50%. The reduction of $A_{33}$, $D_{33}$, and $R_{55}$ varied between 15 and 30%. The $R_{44}$ stiffness reduction was approximately 0% for the non-rotated perforation, while for the rotation angle of 90 degrees, it was about 20%. A very similar stiffness degradation could be observed for the DW model (see Figure 12f).

For samples with different fluting orientations (see Figure 13 and Figure 14), the greatest reduction in stiffness always occurred in the direction perpendicular to the perforation (i.e., ${\left(*\right)}_{22}$ and ${\left(*\right)}_{55})$, regardless of material orientation. Both $A_{22}$ and $D_{22}$ stiffnesses had the greatest reductions and amounted to about 50% in the case of 6/2 mm perforation for all fluting orientations. Slightly smaller reductions in stiffness were observed for $R_{44}$, $A_{33}$, and $D_{33}$ ranging from 15 to 30% (for 6/2 mm perforation type), depending on the orientation of the fluting. The smallest stiffness reductions were observed for $A_{11}$, $D_{11}$, and $R_{55}$.

When analyzing the stiffness reductions for models with shifted fluting (see Figure 9), even in the case without perforation, slight differences in stiffness could be observed (see Table 10 and Table 11) and concerned mainly $R_{44}$ and $R_{55}$. Small fluctuations were also observed in models with perforation for both cases of SW and DW (see Figure 15), where again, the $R_{44}$ and $R_{55}$ showed the greatest dependence on fluting shift.

By also adding to the model the crushing of fluting (see Figure 8) that accompanies the perforations during the treatment of corrugated board, the degradation for some stiffnesses can increase several times (see Figure 16). The more perforated the model (i.e., 6/2 mm perforation type), the smaller the further reductions in the stiffness $A_{22}$, $D_{22}$, and $R_{55}$. The remaining stiffnesses were drastically reduced with the increase in the crushing of the cross-section of the corrugated board. It is worth noting that for the DW model, the stiffnesses reduction of $A_{11}$, $A_{22}$, and $A_{33}$ did not depend on the amount of crushing.

## 5. Conclusions

This article presents the comprehensive numerical analyses of the effect of perforation on reducing stiffness while implementing homogenization techniques. The acquired knowledge can be used for numerical modeling, for example, of corrugated cardboard packaging with perforations. Knowing the specific values of the stiffness reduction, it is possible to correctly model the perforation line and thus accurately estimate the load capacity of the packaging. The reduction in individual stiffnesses depends not only on the type of perforation, but also on the orientation of the perforation and the orientation of the fluting, but does not depend on the location of the perforation along the wavelength. Further development of the launched research is planned related to the validation of the proposed model with experimental models while engaging the non-contact displacement measurements [46].

## Figures and Tables

**Table 1:** Material data of intact double wall corrugated cardboard used for modeling the paper layers according to orthotropic constitutive relation.

**Layers**	$E_{1}\;$	$E_{2}\;$	$\nu_{12}\;$	$G_{12}\;$	$G_{13}\;$	$G_{23}\;$
	(MPa)	(MPa)	(-)	(MPa)	(MPa)	(MPa)
liners	3326	1694	0.34	859	429.5	429.5
fluting	2614	1532	0.32	724	362	362

**Table 2:** Sample symbols.

Perforation Type	Model SW	Model DW
4 mm cut, 4 mm gap	SW-44-Y ^1^-xx ^2^	DW-44-Y-xx
2 mm cut, 6 mm gap	SW-26-Y-xx	DW-26-Y-xx
6 mm cut, 2 mm gap	SW-62-Y-xx	DW-62-Y-xx

^1^ Y means model type and can be: F-flute or C-cut. ^2^ xx is the cut or crease orientation and can be: 00, 15, 30, 45, 60, 75, or 90.

**Table 3:** The stiffnesses of representative shell element computed for a different approach of modeling confronted with data from [39] for saw tooth geometry.

Stiffness	Ref. [39]	Corse Model	Fine Model
$A_{11},\;\left(N/\mathrm{mm}\right)$	1.108 10^6^	1.118 10^6^	1.118 10^6^
$A_{22},\;\left(N/\mathrm{mm}\right)$	1.358 10^6^	1.380 10^6^	1.378 10^6^
$A_{12},\;\left(N/\mathrm{mm}\right)$	3.324 10^5^	3.449 10^5^	3.448 10^5^
$A_{33},\;\left(N/\mathrm{mm}\right)$	4.168 10^5^	4.115 10^5^	4.115 10^5^
$D_{11},\;\left(N\cdot \mathrm{mm}\right)$	9.195 10^8^	9.211 10^8^	9.210 10^8^
$D_{22},\;\left(N\cdot \mathrm{mm}\right)$	9.822 10^8^	9.926 10^8^	9.925 10^8^
$D_{12},\;\left(N\cdot \mathrm{mm}\right)$	2.758 10^8^	2.777 10^8^	2.777 10^8^
$D_{33},\;\left(N\cdot \mathrm{mm}\right)$	3.220 10^8^	3.269 10^8^	3.268 10^8^
$A_{44}$, (N/mm)	-	5.194 10^4^	5.184 10^4^
$A_{55}$, (N/mm)	-	7.408 10^4^	7.376 10^4^

**Table 4:** Constitutive stiffness matrix $A_{k}$ for the SW model without perforation.

		A & B			B & D			R	
		1	2	3	1	2	3	4	5
**A & B**	1	2184.4	388.92	0	0	0	0		
	2	388.92	1756.9	0	0	0	0		
	3	0	0	667.81	0	0	0		
**B & D**	1	0	0	0	8628.2	1506.5	0		
	2	0	0	0	1506.5	5469.3	0		
	3	0	0	0	0	0	2300.2		
**R**	4							105.08	0
	5							0	130.91

**Table 5:** Constitutive stiffness matrix $A_{k}$ for the DW model without perforation.

		A & B			B & D			R	
		1	2	3	1	2	3	4	5
**A & B**	1	3313.8	593.33	0	1117.1	195.90	0		
	2	593.33	2967.5	0	196.36	1200.6	0		
	3	0	0	1077.8	0	0	409.89		
**B & D**	1	1117.1	196.36	0	20 619	3620.8	0		
	2	195.90	1200.6	0	3620.8	15 042	0		
	3	0	0.0	409.89	0	0	5934.5		
**R**	4							233.13	0
	5							0	242.28

**Table 6:** Selected stiffnesses in SW samples with no perforation and with different flute orientations.

	SW-0-F-00	SW-0-F-15	SW-0-F-30	SW-0-F-45	SW-0-F-60	SW-0-F-75	SW-0-F-90
$A_{11}$ (MPa mm)	2184.4	2127.2	1990.3	1854.2	1774.2	1751.5	1756.9
$A_{22}$ (MPa mm)	1756.9	1751.5	1774.2	1854.2	1990.3	2127.2	2184.4
$A_{33}$ (MPa mm)	667.81	699.26	760.50	792.80	760.50	699.30	667.80
$D_{11}$ (MPa mm^3^)	8628.2	8313.5	7480.9	6521.5	5897.3	5575.8	5469.3
$D_{22}$ (MPa mm^3^)	5469.3	5575.8	5897.3	6520.4	7480.9	8313.5	8628.2
$D_{33}$ (MPa mm^3^)	2300.2	2425.2	2650.1	2755.4	2650.1	2425.2	2300.2
$R_{44}$ (MPa mm)	105.08	108.15	119.80	132.90	127.20	126.20	130.90
$R_{55}$ (MPa mm)	130.91	126.16	127.20	132.80	119.80	108.10	105.10

**Table 7:** Selected stiffnesses in DW samples with no perforation and with different flute orientations.

	DW-0-F-00	DW-0-F-15	DW-0-F-30	DW-0-F-45	DW-0-F-60	DW-0-F-75	DW-0-F-90
$A_{11}$ (MPa mm)	3313.8	3250.6	3090.4	2955.2	2912.0	2939.7	2967.5
$A_{22}$ (MPa mm)	2967.5	2939.7	2912.0	2955.3	3090.4	3250.6	3313.8
$A_{33}$ (MPa mm)	1077.8	1127.5	1225.3	1275.9	1225.3	1127.5	1077.8
$D_{11}$ (MPa mm^3^)	20,242	19,610	17,980	16,221	15,123	14,662	14,556
$D_{22}$ (MPa mm^3^)	14,556	14,662	15,123	16,220	17,980	19,610	20,242
$D_{33}$ (MPa mm^3^)	5778.6	6071.8	6634.3	6910.6	6634.3	6071.8	5778.6
$R_{44}$ (MPa mm)	233.13	240.21	246.71	257.56	247.51	242.88	242.28
$R_{55}$ (MPa mm)	242.28	242.88	247.51	257.43	246.71	240.21	233.13

**Table 8:** The selected stiffnesses in SW models for different perforations and flute rotated by 15 degrees.

Stiffness	SW-0-F-15	SW-26-F-15	SW-44-F-15	SW-62-F-15
$A_{11}$ (MPa mm)	2127.2	2116.1	2082.1	2052.3
$A_{22}$ (MPa mm)	1751.6	1609.1	1267.7	885.12
$A_{33}$ (MPa mm)	699.26	681.92	608.30	524.18
$D_{11}$ (MPa mm^3^)	8313.4	8276.1	8166.4	8048.5
$D_{22}$ (MPa mm^3^)	5575.8	5290.9	4291.8	2877.2
$D_{33}$ (MPa mm^3^)	2425.2	2384.5	2216.7	1968.9
$R_{44}$ (MPa mm)	108.15	107.68	106.48	106.77
$R_{55}$ (MPa mm)	126.16	120.04	94.100	83.465

**Table 9:** Stiffness reduction for both SW and DW samples with flute rotated by 15 degrees for three cases of perforation.

Stiffness Reduction	SW-26-F-15 (%)	SW-44-F-15 (%)	SW-62-F-15 (%)	DW-26-F-15 (%)	DW-44-F-15 (%)	DW-62-F-15 (%)
$1-A_{11}$ $/A_{11}^{*}$	0.523	2.121	3.519	0.508	1.903	3.364
$1-A_{22}$ $/A_{22}^{*}$	8.133	27.66	49.46	7.852	27.77	50.98
$1-A_{33}$ $/A_{33}^{*}$	2.480	13.01	25.04	2.735	12.66	24.50
$1-D_{11}$ $/D_{11}^{*}$	0.449	1.769	3.187	0.467	1.786	3.247
$1-D_{22}$ $/D_{22}^{*}$	5.110	23.03	48.40	6.377	25.41	49.18
$1-D_{33}$ $/D_{33}^{*}$	1.677	8.598	18.81	2.171	10.25	20.88
$1-R_{44}$ $/R_{44}^{*}$	0.435	1.545	1.273	−0.349	1.032	1.177
$1-R_{55}$ $/R_{55}^{*}$	4.851	25.41	33.84	4.060	18.48	30.95

* denotes the reference value of non-perforated specimen (i.e., SW-0-F-15).

**Table 10:** Uncut samples SW. Stiffness reduction in terms of flute offset.

Stiffness Reduction	1/16 P (%)	2/16 P (%)	3/16 P (%)	4/16 P (%)
$1-A_{11}$ $/A_{11}^{*}$	−0.023	−0.121	−1.061	−0.055
$1-A_{22}$ $/A_{22}^{*}$	−0.018	−0.061	−0.086	−0.003
$1-A_{33}$ $/A_{33}^{*}$	−0.035	−0.089	−0.062	0.038
$1-D_{11}$ $/D_{11}^{*}$	0.023	0.099	−0.687	0.059
$1-D_{22}$ $/D_{22}^{*}$	0.018	0.053	−0.007	0.050
$1-D_{33}$ $/D_{33}^{*}$	0.124	0.495	1.102	1.720
$1-R_{44}$ $/R_{44}^{*}$	3.533	13.41	10.63	1.771
$1-R_{55}$ $/R_{55}^{*}$	1.286	4.036	8.186	8.956

* denotes the reference value of non-shifted flute.

**Table 11:** Uncut samples DW. Stiffness reduction in terms of flute offset.

Stiffness Reduction	1/16 P (%)	2/16 P (%)	3/16 P (%)	4/16 P (%)
$1-A_{11}$ $/A_{11}^{*}$	−0.018	−0.094	−1.052	−0.037
$1-A_{22}$ $/A_{22}^{*}$	−0.013	−0.044	−0.075	−0.003
$1-A_{33}$ $/A_{33}^{*}$	−0.032	−0.082	−0.056	0.039
$1-D_{11}$ $/D_{11}^{*}$	0.012	0.029	−1.048	−0.012
$1-D_{22}$ $/D_{22}^{*}$	0.011	0.009	−0.062	0.021
$1-D_{33}$ $/D_{33}^{*}$	−0.029	0.110	0.459	0.880
$1-R_{44}$ $/R_{44}^{*}$	2.706	9.932	8.977	1.396
$1-R_{55}$ $/R_{55}^{*}$	2.378	6.572	11.88	15.28

* denotes the reference value of non-shifted flute.

## Data Availability

The data presented in this study are available on request from the corresponding author.

## References

[1] Sohrabpour V., Hellström D. Models and software for corrugated board and box design. Proceedings of the 18th International Conference on Engineering Design (ICED 11).

[2] Kellicutt K., Landt E. (1952). Development of design data for corrugated fiberboard shipping containers. Tappi J..

[3] Maltenfort G. (1956). Compression strength of corrugated containers. Fibre Contain..

[4] McKee R.C., Gander J.W., Wachuta J.R. (1963). Compression strength formula for corrugated boxes. Paperboard Packag..

[5] Allerby I.M., Laing G.N., Cardwell R.D. (1985). Compressive strength—From components to corrugated containers. Appita Conf. Notes.

[6] Schrampfer K.E., Whitsitt W.J., Baum G.A. (1987). Combined Board Edge Crush (ECT) Technology.

[7] Batelka J.J., Smith C.N. (1993). Package Compression Model.

[8] Urbanik T.J., Frank B. (2006). Box compression analysis of world-wide data spanning 46 years. Wood Fiber Sci..

[9] Avilés F., Carlsson L.A., May-Pat A. (2012). A shear-corrected formulation of the sandwich twist specimen. Exp. Mech..

[10] Garbowski T., Gajewski T., Grabski J.K. (2020). The role of buckling in the estimation of compressive strength of corrugated cardboard boxes. Materials.

[11] Garbowski T., Gajewski T., Grabski J.K. (2021). Estimation of the compressive strength of corrugated cardboard boxes with various openings. Energies.

[12] Garbowski T., Gajewski T., Grabski J.K. (2021). Estimation of the compressive strength of corrugated cardboard boxes with various perforations. Energies.

[13] Wang L., Qian Z. (2020). A meshfree stabilized collocation method (SCM) based on reproducing kernel approximation. Comput. Methods Appl. Mech. Eng..

[14] Wang L., Liu Y., Zhou Y., Yang F. (2021). Static and dynamic analysis of thin functionally graded shell with in-plane material inhomogeneity. Int. J. Mech. Sci..

[15] Chu F., He J., Wang L., Zhong Z. (2016). Buckling analysis of functionally graded thin plate with in-plane material inhomogeneity. Eng. Anal. Bound. Elem..

[16] Frank B. (2014). Corrugated box compression—A literature survey. Packag. Technol. Sci..

[17] Stott R.A. (2017). Compression and stacking strength of corrugated fibreboard containers. Appita J..

[18] Junli W., Quancheng Z. (2006). Effect of moisture content of corrugated box on mechanical properties. J. Lanzhou Jiaotong Univ..

[19] Archaviboonyobul T., Chaveesuk R., Singh J., Jinkarn T. (2020). An analysis of the influence of hand hole and ventilation hole design on compressive strength of corrugated fiberboard boxes by an artificial neural network model. Packag. Technol. Sci..

[20] Zhang Y.-L., Chen J., Wu Y., Sun J. (2011). Analysis of hazard factors of the use of corrugated carton in packaging low-temperature yogurt during logistics. Procedia Environ. Sci..

[21] Gallo J., Cortés F., Alberdi E., Goti A. (2021). Mechanical behavior modeling of containers and octabins made of corrugated cardboard subjected to vertical stacking loads. Materials.

[22] Thakkar B.K., Gooren L.G.J., Peerlings R.H.J., Geers M.G.D. (2008). Experimental and numerical investigation of creasing in corrugated paperboard. Philos. Mag..

[23] Beex L.A.A., Peerlings R.H.J. (2009). An experimental and computational study of laminated paperboard creasing and folding. Int. J. Solids Struct..

[24] Giampieri A., Perego U., Borsari R. (2011). A constitutive model for the mechanical response of the folding of creased paperboard. Int. J. Solids Struct..

[25] Domaneschi M., Perego U., Borgqvist E., Borsari R. (2017). An industry-oriented strategy for the finite element simulation of paperboard creasing and folding. Pack. Technol. Sci..

[26] Awais M., Tanninen P., Leppänen T., Matthews S., Sorvari J., Varis J., Backfol K. (2018). A computational and experimental analysis of crease behavior in press forming process. Procedia Manuf..

[27] Leminena V., Tanninena P., Pesonena A., Varis J. (2019). Effect of mechanical perforation on the press-forming process of paperboard. Procedia Manuf..

[28] Nordstrand T. (2003). Basic Testing and Strength Design of Corrugated Board and Containers. Ph.D. Thesis.

[29] Nordstrand T., Carlsson L. (1997). Evaluation of transverse shear stiffness of structural core sandwich plates. Comp. Struct..

[30] Garbowski T., Gajewski T., Grabski J.K. (2020). Role of transverse shear modulus in the performance of corrugated materials. Materials.

[31] Garbowski T., Gajewski T., Grabski J.K. (2020). Torsional and transversal stiffness of orthotropic sandwich panels. Materials.

[32] Garbowski T., Gajewski T. (2021). Determination of transverse shear stiffness of sandwich panels with a corrugated core by numerical homogenization. Materials.

[33] Urbanik T.J., Saliklis E.P. (2003). Finite element corroboration of buckling phenomena observed in corrugated boxes. Wood Fiber Sci..

[34] Garbowski T., Jarmuszczak M. (2014). Homogenization of corrugated paperboard. Part 1. Analytical homogenization. Pol. Pap. Rev..

[35] Garbowski T., Jarmuszczak M. (2014). Homogenization of corrugated paperboard. Part 2. Numerical homogenization. Pol. Pap. Rev..

[36] Marek A., Garbowski T. (2015). Homogenization of sandwich panels. Comput. Assist. Methods Eng. Sci..

[37] Garbowski T., Marek A. Homogenization of corrugated boards through inverse analysis. Proceedings of the 1st International Conference on Engineering and Applied Sciences Optimization.

[38] Hohe J. (2003). A direct homogenization approach for determination of the stiffness matrix for microheterogeneous plates with application to sandwich panels. Compos. Part B.

[39] Buannic N., Cartraud P., Quesnel T. (2003). Homogenization of corrugated core sandwich panels. Comp. Struct..

[40] Biancolini M.E. (2005). Evaluation of equivalent stiffness properties of corrugated board. Comp. Struct..

[41] Abbès B., Guo Y.Q. (2010). Analytic homogenization for torsion of orthotropic sandwich plates: Application to corrugated cardboard. Comp. Struct..

[42] Ramírez-Torres A., Penta R., Rodríguez-Ramos R., Merodio J., Sabina F.J., Bravo-Castillero J., Guinovart-Díaz R., Preziosi L., Grillo A. (2018). Three scales asymptotic homogenization and its application to layered hierarchical hard tissues. Int. J. Solids Struct..

[43] Ramírez-Torres A., Di Stefano S., Grillo A., Rodríguez-Ramos R., Merodio J., Penta R. (2018). An asymptotic homogenization approach to the microstructural evolution of heterogeneous media. Int. J. Non-Lin. Mech..

[44] Garbowski T., Gajewski T., Mrówczyński D., Jędrzejczak R. (2021). Crushing of single-walled corrugated board during converting: Experimental and numerical study. Energies.

[45] Gajewski T., Garbowski T., Staszak N., Kuca M. (2021). Crushing of double-walled corrugated board and its influence on the load capacity of various boxes. Preprints.

[46] Garbowski T., Grabski J.K., Marek A. (2021). Full-field measurements in the edge crush test of a corrugated board—Analytical and numerical predictive models. Materials.

